# MicroRNA in Metabolic Re-Programming and Their Role in Tumorigenesis

**DOI:** 10.3390/ijms17050754

**Published:** 2016-05-18

**Authors:** Marco Tomasetti, Monica Amati, Lory Santarelli, Jiri Neuzil

**Affiliations:** 1 Department of Clinical and Molecular Sciences, Polytechnic University of Marche, Ancona 60020, Italy; m.amati@univpm.it (M.A.); l.santarelli@univpm.it (L.S.); 2 Mitochondria, Apoptosis and Cancer Research Group, School of Medical Science and Menzies Health Institute Queensland, Griffith University, Southport, QLD 4222, Australia; 3 Molecular Therapy Group, Institute of Biotechnology, Czech Academy of Sciences, Prague-West 25243, Czech Republic

**Keywords:** miRNAs, metabolic reprogramming, tumorigenesis, miR-126 and cancer-stroma environment, miRNA regulating signaling pathways

## Abstract

The process of metabolic re-programing is linked to the activation of oncogenes and/or suppression of tumour suppressor genes, which are regulated by microRNAs (miRNAs). The interplay between oncogenic transformation-driven metabolic re-programming and modulation of aberrant miRNAs further established their critical role in the initiation, promotion and progression of cancer by creating a tumorigenesis-prone microenvironment, thus orchestrating processes of evasion to apoptosis, angiogenesis and invasion/migration, as well metastasis. Given the involvement of miRNAs in tumour development and their global deregulation, they may be perceived as biomarkers in cancer of therapeutic relevance.

## 1. Introduction

Cancer metabolism is the result of a combination of genetic instability and flexibility to adapt to the microenvironment [1]. The uncontrolled cell growth is one of the hallmarks of cancer disease, which involves deregulated pathway signaling, in association with adjustment of energy metabolism and energy production. Aerobic glycolysis, glutaminolysis and altered autophagy represent the most adaptive metabolic mechanisms of cancer cells. These bioenergetics and metabolic features have been associated with activated oncogenes, such as RAS, MYC and p53, whose alterations allow cancer cells to proliferate and survive under adverse conditions. For instance, the insulin/insulin-like growth factor-I receptor (IGF-IR) signaling cascade activates the PI3/Akt pathway, which plays a central role in regulating survival and apoptosis, proliferation, protein and lipid synthesis and glucose metabolism [2]. Akt promotes the mitochondrial extrusion of citrate from the tricarboxylic acid (TCA) cycle to produce acetyl-CoA by activation of ATP citrate lyase (ACL) [3]. ACL links glucose metabolism to lipid synthesis, and its inhibition arrests tumour growth and diminishes Akt-induced tumorigenesis [4]. The cross-talk between epithelial cancer cells and the stromal compartment and the metabolic coupling between well-oxygenated and hypoxic compartments confer to cancer cells the ability to switch from oxidative phosphorylation to aerobic glycolysis and *vice versa*. The stromal compartment actively contributes to tumour formation and growth. Among the stromal components, cancer-associated fibroblasts (CAFs) and endothelia cells (EC) have recently taken a pivotal stage [5]. Cancer cells in response to stromal cells contact enhance their invasiveness, the intra- and extravasation and their stemness.

MicroRNAs (miRNAs) have emerged as key players in the tumour microenvironment, being involved in the development of cancer and its progression. miRNAs may be relevant in the fine tuning of adaptation to stress situations, such as oncogenic events, hypoxia, nutrient deprivation and oxidative stress [6]. Therefore, the emerging role of miRNAs within a tumour microenvironment is likely of paramount importance. Recent findings showed that miRNAs can be found inside exosomes and may mediate the cross-talk between cancer cells and the surrounding cells. This review will focus on the role of miRNAs in metabolic re-programming associated with cancer and their involvement in the cross-talk between cellular and stromal components of the tumour microenvironment. Advances in understanding the underlying biology of tumour-stroma interactions and tumour miRNAs are critical to guiding rational approaches to designing novel treatment strategies.

## 2. Tumour Environment and Cancer Metabolic Reprogramming

Cancer cells exert increased metabolic plasticity that allows them to continuously adapt to changes in the tumour environment. In this context, cancer cells closely interact with neighbouring stromal cells, and together, they cooperatively promote pathology via bidirectional communication. The high rate of glycolysis and suppressed gluconeogenesis help to maintain a low level of intracellular glucose, creating a gradient favouring the flux of glucose into the cell and create microenvironmental acidosis, compelling the evolution of phenotypes with stress resistance and metastatic capacity. These alterations enable cancer cells to acquire characteristics, such as resistance to growth inhibitory factors, proliferation, evasion of apoptosis, abnormal angiogenesis and evasion of destruction by the immune system. These hallmarks of cancer are intertwined with altered intrinsic cellular metabolism. Such metabolic re-programming is linked to the activation of oncogenes and/or suppression of tumour suppressor genes, which are further regulated by either their activation or inactivation dynamics, where miRNAs appear to play important roles [7]. Evidence revealing the involvement of miRNAs in the regulation of essential cellular homeostasis pathways suggests that miRNAs can be critically involved in tumorigenesis and cancer progression [8]. It is increasingly becoming clear that metabolic re-programming plays a critical role in tumorigenesis and metastasis.

### 2.1. miRNA Regulate Glucose Metabolism

The most prominent metabolic alteration in cancer cells is increased glucose uptake and the use of glycolysis rather than mitochondrial respiration regardless of the presence of oxygen [9]. miRNAs can regulate intracellular glucose levels by modulating the transcription and expression of glucose transporters. Glucose transporters (GLUTs) are responsible for the intake of glucose by cancer cells along the concentration gradient. miR-195-5p, which has been found downregulated in bladder cancer, targets GLUT-3; miR-223, miR-93 and miR-133 target the insulin-sensitive glucose transporter GLUT-4, [10,11]. The hexokinase 2 (HK2) glycolytic enzyme is highly expressed in tumours, and miR-143, which targets HK2, has been found downregulated in cancer cells [12]. miR-155 represses miR-143, thus resulting in upregulation of HK2 at the post-transcriptional level [13]. Subsequent experiments of cellular functions demonstrated that miR-143 acts as a tumour suppressor by affecting cell viability, apoptosis and the cell cycle of prostate cancer [14]. The miR-15a/16-1 cluster downregulates aldolase A (Aldo A), a glycolytic enzyme involved in the breakdown of fructose 1,6-bisphosphate into glyceraldehyde 3-phosphate and dihydroxyacetone phosphate [15]. Aldo A is a direct target of miR-122 in liver cells [16]. In addition, miR-1 and miR-206 were found to regulate glycerol-3-phosphate dehydrogenase-2 (GPD2) [17].

### 2.2. miRNAs Regulate the Tricarboxylic Acid Cycle

Metabolic intermediates produced by glycolytic flux nourish biosynthetic pathways to synthesize nucleotides and amino acids. Glutamine is the main carbon source for the replenishment of TCA cycle intermediates. The enzyme glutaminase (GLS) generates glutamate from glutamine, which enters into the TCA cycle as α-ketoglutarate to produce cellular energy. miR-23 was found to target GLS [18]. Glutamine-derived citrate can be converted to acetyl-CoA (AcCoA) and oxaloacetate (OAA) by ATP citrate lyase (ACL). AcCoA feeds into *de novo* fatty acid (FA) synthesis, and OAA can be metabolized to nucleotides and amino acids [19]. These events drive the biosynthesis of the macromolecules and organelles required for cell proliferation. Conceivably, two populations have been found in tumours; a glucose-dependent population that releases lactate and a lactate-dependent population, which utilizes lactate produced by their surrounding cells [20]. These two populations symbiotically sustain each other. In hypoxic conditions that operate within the stroma of tumour, cancer cells use glucose for fuel and secrete lactate as a metabolite, which is used by the other cells. The hypoxia response system acts by upregulating glucose transporters and enzymes involved in the glycolytic pathway [21]. miR-34, let-7 and miR-107 regulate glycolysis by targeting lactate dehydrogenase A (LDHA) via p53 [22].

It was predicted that miR-103 and miR-107 control AcCoA and the level of lipids by upregulating pantothenate kinase (PanK), the first enzyme in the coenzyme A biosynthetic pathway that phosphorylates pantothenate to form 4′-phosphopantothenate [23]. Other miRNAs were found to control the expression of metabolically-associated genes. For instance, a set of miRNAs, miR-152, miR-148a, miR-494 and miR-19a, regulate the expression of citrate synthase genes. Additionally, miR-181a and miR-183 target isocitrate dehydrogenase 1/2 (IDH1/2), enzymes of the TCA cycle [24,25]. Additionally, miR-210 controls the TCA cycle by targeting iron-sulphur cluster assembly proteins (ISCU1/2), cytochrome c oxidase 10 (COX10), succinate dehydrogenase, subunit D (SDHD) or ADH dehydrogenase (ubiquinone) 1 α subcomplex, 4 (NDUFA4) in several cancers [26]. miR-378* also regulates the TCA cycle in breast cancer by inhibiting the expression of PGC-1β (peroxisome proliferator-activated receptor α, co-activator 1-α), ERRγ (estrogen-related receptor α) and GABPA (GA binding protein transcription factor, α subunit) [27]. miR-23a-mediated suppression of PGC-1α could also facilitate the metabolic shift from OXPHOS to anaerobic glycolysis to synthesise anabolic precursors to sustain proliferation of tumour cells [28]. On the other hand, over-expression of miR-125b results in repression of many transcripts encoding enzymes implicated in glucose, glutathione (GSH) and lipid metabolism, including PDK1 (pyruvate dehydrogenase kinase 1) [29]. miR-26a inhibits the expression of pyruvate dehydrogenase protein X, a non-catalytic subunit of the pyruvate dehydrogenase (PDH) complex, which efficiently decrease the process of pyruvate acetyl-CoA conversion and, thus, blocks the key rate-limiting step of glycolysis to the TCA cycle as a part of glucose metabolism, impairing mitochondrial metabolism [30].

The pyruvate kinases M1 (PKM1) and M2 (PKM2) isoform ratio also controls the process of glycolysis. While PKM2 is expressed in embryonic, proliferating and cancer cells and promotes glycolysis, PKM1 is expressed in normal differentiated tissue and promotes OXPHOS. PKM isoforms are targeted by miR-124, miR-137 and miR-340; therefore, these miRNAs impair cancer growth by counteracting Warburg’s effect by modulating the PKM isoform ratio [31]. Furthermore, miRNAs also regulate the TCA cycle indirectly by acting on the transcription factors MYC and HIF. MYC is one of the major regulators of glutaminolysis; therefore, the notion of its regulation by miRNAs supports the concept that MYC promotes not only cell proliferation, but also the process of the production of various macromolecules and antioxidants (GSH) that are required for efficient growth.

Although aerobic glycolysis is a hallmark of cancer, a wide variety of tumours rely in mitochondrial metabolism by triggering adaptive mechanisms to optimise their oxidative phosphorylation in relation to their substrate supply and energy demands. Horizontal transfer of mitochondrial DNA (mtDNA) from host cells to tumour cells with compromised respiratory function within the tumour microenvironment has been observed for breast carcinoma cells. These cells lacking mtDNA re-established respiration and tumour-initiating efficacy [32]; thus supporting the notion of the high plasticity of malignant cells able to overcome mtDNA damage through pathophysiological mechanisms. Mitochondria are involved in various functions in cancer cells to promote tumour growth and survival in response to stress [33]. Recently, a study aimed at evaluating the miRNAs’ translocation from the nuclei to mitochondria and their implication in the mitochondrial function found that miR-181c translocates into mitochondria and targets COX1 (cytochrome c oxidase subunit 1), leading to re-modelling of the complex IV of the electron transfer chain (ETC) with ensuing mitochondrial dysfunction [34]. In another study, it was hypothesised that miR-338 regulates mitochondrial OXPHOS by directly targeting COXIV (cytochrome c oxidase subunit IV) [35].

### 2.3. miRNAs and Lipid Metabolism

Cancer cells show an altered lipid metabolism, where fatty acid (FAs) are mainly synthesised *de novo* [36]. Mitochondrial activity within the pyruvate-citrate shuttle is critical for the synthesis of FAs and cholesterol, as well as protein acetylation. Mitochondrial fatty acid oxidation (FAO) provides an alternative pathway to support cancer cell survival in glucose-limiting conditions by producing AcCoA in each cycle. AcCoA can enter the TCA cycle to produce NADH and FADH2 to fuel the ETC for ATP production. Alternatively, AcCoA condenses with OAA to produce citrate that can be further redirected to NADPH, generating reactions in the cytoplasm via IDH1 and malic enzyme activity [37]. miR-33a/b are known players that affect the expression of cholesterol and lipid metabolism, as well as energy homeostasis by regulating the level of cholesterol efflux, such as ABCA proteins, and AMPKa1, SIRT6 and insulin receptor substrate 2 (IRS2) [38]. miR-122 acts as an important regulator of cholesterol and FA metabolism [39]. miR-122 has been described to stimulate the production of endoplasmic reticulum (ER)-associated lipid droplets (LDs) and cholesterol-rich membrane domains [40]; inhibition of miR-122 may contribute a shift in the equilibrium between lipid storage and metabolism [41].

**Figure 1:**
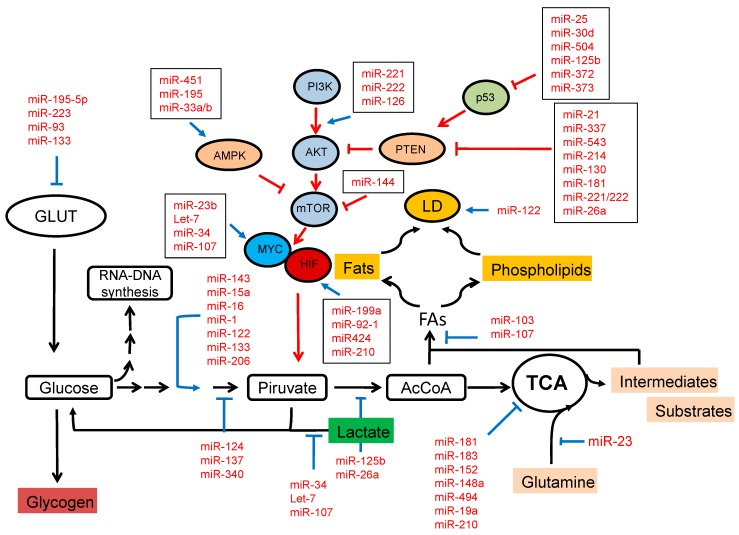
miRNA network regulating cancer metabolism. The shift to aerobic glycolysis in cancer cells may be driven or enhanced by miRNAs via regulation of signaling pathways and/or transcription factors. miRNAs could regulate cell metabolism by modulating the expression of glucose transporters (GLUTs), enzymes involved in glycolysis, the TCA cycle and lipid synthesis plus their storage in lipid droplets (LDs). The activation of the PI3K/AKT/mTOR pathway or HIF-and MYC-dependent transcription by means of the suppression of inhibitory miRNAs or the upregulation of oncogenic miRNAs supports increased glycolysis and inhibits the TCA cycle. Conversely, AMPK signaling inhibits mTOR, and p53 suppresses glycolysis and increases mitochondrial metabolism.

FAs and their derivatives are rapidly incorporated into LDs in “well-fed” cells, but transported from LDs into mitochondria when cells undergo starvation [42,43]. LDs are complex and dynamic “organelles” (storage vehicles) that store and supply lipids for energy metabolism, membrane synthesis and the production of essential lipid-derived molecules [44]. LD biogenesis initiates with the esterification of AcCoA with glycerol-3-phosphate to generate lysophosphatidic acid (LPA). LPA is then converted into phosphatidic acid (PA), giving rise to diacylglycerol (DAG) after its dephosphorylation. Diacylglycerol acyltransferase (DGAT) adds AcCoA to generate triacylglycerol (TAG). miRNAs might be directed to inhibit LD biogenesis, a process that could have consequences for the accommodation of increased lipogenesis and additional regulation of its functions. On the other hand, miRNA-mediated inhibition of lipid mobilisation may promote cell death due to decreased availability of lipids for membrane synthesis, signaling and energy production. A specific function of autophagy in lipid mobilisation that may have important consequences in the regulation of cellular lipid content has been recently described [45]. LDs have a regulatory role by means of their interaction with intracellular compartments to provide specific lipids or sequestering misfolded and functional proteins. The miR-network involved in regulating pathways in cancer metabolism is shown in Figure 1.

### 2.4. Regulation of Signalling Pathways by miRNAs

The process of metabolic re-programming is linked to the activation of oncogenes and/or oncosuppressor genes, which are further regulated by miRNAs. Accordingly, the interplay between deregulated miRNAs and impaired signaling pathways mainly contributes to an altered metabolism. The principal pathways involved in the re-programming include HIF, MYC, p53, AMPK, AKT and insulin signaling. The hypoxia-inducible factor (HIF) acts as a transcription factor regulating gene expression under oxygen deprivation. HIF induces glycolytic shift via activation of genes encoding glucose transporters and glycolytic enzymes and reinforces the glycolytic phenotype by activation of PDKs, which reduce the flow of pyruvate into the TCA cycle [46]. miR-199a downregulation upon hypoxia is required for rapid upregulation of its target, HIF1α [47]. Stabilisation of HIF1α under normoxia is mediated by miR-92-1 that targets the von Hippel–Lindau (VHL) tumour suppressor [48], an E3 ubiquitin ligase, which binds to HIF1α, thus inducing its degradation in the presence of oxygen. Several other miRNAs, whose levels are altered in multiple cancers, such as miR-20a/b, miR-21, miR-22, miR-101, miR-106a/b, miR-150 and miR-200b, are predicated to target VHL [49]. miR-424 upregulation in endothelial cells stabilises HIF1α by targeting cullin 2, a scaffolding protein involved in assembling the ubiquitin lyase system [50]. miR-210 defined as hypoxic miRNA induces HIF1α stabilisation by directly targeting glycerol-3-phosphate dehydrogenase 1-like (GPD1L) protein, an inhibitor of HIF1α [51].

The oncogenic transcription factor MYC, in concert with HIF1α, regulates the activation of glucose transporters, glycolytic enzymes, LDHA and PDK1, and has a crucial role in glutamine metabolism mediated by miR-23b [52]. Let-7a regulates c-MYC mRNA, and its overexpression can inhibit the growth of lung cancer grafts transplanted subcutaneously in nude mice [53]. Overexpression of let-7 results in insulin resistance and impaired glucose tolerance through the let-7-mediated repression of insulin/PI3K/Akt signaling [54]. On the other hand, c-MYC transcriptionally represses miR-23a and miR-23b, resulting in increased expression of mitochondrial glutaminase, enhancing glutamine catabolism [52]. MYC is also known to induce another important oncogenic miRNA, miR-9, which has been implicated in tumour cell invasiveness and metastasis [55]. Further, p53 regulates several miRNAs that control metabolism in cancer; it also modulates the expression of miR-34, the miR-194/miR-215 cluster, let-7 and miR-107, which further inhibits the expression of their target genes, including LDHA, MYC and sirtuin-1 (SIRT1), as well as HIF [22,56,57]. p53 suppresses the transcription of some tumorigenic miRNAs, such as the miR-17-92 cluster, miR-16-1, miR-143 and miR-145, involved in controlling cell proliferation through cell cycle arrest by targeting KRAS and CDK6 [58,59]. miR-25, miR-30d, miR-504, miR-125b, miR-372 and miR-373 directly target p53 3’-UTR and hence impair p53 response and promote tumorigenicity [60,61].

The AMP-activated protein kinase (AMPK) is activated during metabolic stress to increase energy conservation and glucose uptake, allowing cells to survive in conditions of low energy supply. It has been reported that PKM2 is a target of miR-326 and that knocking down PKM2 decreased ATP and GSH levels, resulting in activation of AMPK [62]. Similarly, miR-451 controls AMPK signaling in glioma cells, and it was found to regulate the LKB1/AMPK pathway by targeting calcium binding protein 9 (CAB9) in association with miR-195 [63]. miR-33a/b have been shown to control the expression of AMPK and SIRT6, which are involved in the regulation of lipid and glucose metabolism. miR-33 targets insulin receptor substrate-2 (IRS2), an adaptor factor that controls insulin signaling, thereby affecting the Akt and forkhead box protein O1 (FOXO1) pathway [38].

The PI3K/AKT pathway, often activated in human cancers, is regulated by several miRNAs [64]. The oncogenic miR-21, miR-337, miR-543, miR-214 and miR-130 are known to induce tumour-associated neovascularisation by directly targeting PTEN and activating PI3K/AKT, as well the ERK1/2 signaling pathways, which increases HIF1α and vascular endothelial growth factor (VEGF) expression [65,66,67,68,69]. The expression of miR-181a was found to be enhanced in cancer cells, which induce a metabolic shift by inhibiting the expression of PTEN, leading to an increase in phosphorylated Akt [70]. Further, miR-26a is also a direct regulator of PTEN, where its loss has been associated with deregulated Akt activity, thus with metastasis and angiogenic potential [71].

A negative feedback mechanism of the PI3K/AKT pathway involves the mTOR kinase, which actively coordinates cell growth and metabolism. mTOR activation inhibits the PI3K pathway, thus increasing the activity of its effector Akt [72]. In this context, miR-144 targets mTOR, thus inhibiting cell proliferation by inducing cell cycle arrest [73]. The signaling pathway PI3K/AKT/mTOR kinase also regulates apoptosis and autophagy in dependence of survival signaling. In conditions of insufficient energy, PI3K/AKT/mTOR kinase is inhibited, resulting in apoptosis/autophagy induction [74]. Interestingly, the miR-221/222 gene cluster, an activator of PI3K/AKT, has been found to induce the process of angiogenesis [75]. Conversely, miR-126 is known to maintain vascular integrity and to inhibit tumour angiogenesis via regulation of VEGF signaling [76]. Both glucose and hypoxia are reported to downregulate miR-126 in the tumour environment [77]. Suppression of miR-126 upregulates the pro-angiogenic factors and causes invasive growth of cervical cancer [78].

PI3K/AKT is a biochemical regulator that functions as an important cross-talk node between several signaling pathways in the mammalian cell. In particular, AKT is a key mediator of glucose transport in response to insulin. The insulin/IGF-IR signaling (IIS) pathway regulates cellular glucose uptake and glycolysis. It is known that glycolysis feeds cancer cells by producing metabolic intermediates, which are used as building blocks for cancer growth. Insulin secretion from pancreatic β cells plays a central role in the control of glucose levels, and miRNAs are recognized as important regulators of β cell survival, proliferation and function. Pancreatic β cells express a specific set of miRNAs, including miR-7, miR-375 and let-7 family members, which affect the cellular composition of the pancreatic islets. For instance, miR-375, which is highly expressed in pancreatic islets, is required for normal glucose homeostasis. Knockout of miR-375 induces hyperglycaemia and hyperglucagonaemia, leading to a diabetic state if crossed with obesity and insulin-resistance [79]. Insulin resistance was found to be positively associated with elevated circulating miR-122 [80]. miR-122 is a liver-specific anti-proliferative miRNA that was found to be transferred via exosomes. The discovery of circulating miRNAs in exosomes emphasizes their importance in endocrine signaling.

Deregulation of miR-122, as major regulator of lipid metabolism in liver, has been related to obesity, metabolic syndrome [41] and non-alcoholic fatty liver diseases (NAFLD), which is characterized by fat accumulation in the liver without significant alcohol consumption [81,82]. It was reported that exosomal miR-122, expressed and released by hepatoma Huh7 cells and taken by their adjacent miR-122-deficient hepatoma HepG2 cells, was found to be effective in the repression of target miRs and to reduce the growth and proliferation of recipient HepG2 cells. Interestingly, in a reciprocal process, HepG2 secretes IGF-I, which in turn inhibits miR-122 biogenesis in neighbouring Huh7 cells. This reciprocal effect exerted by HepG2 on miR-122-producing neighbouring cells may indicate a strategy that hepatic cancer cells adopt to modulate their microenvironment to their benefit and proliferation [83]. The package of miR-122 into secreted exosomes, which mediate miR-122 cell to cell communication, further increases the sensitivity of human hepatocellular carcinoma (HCC) cells to chemotherapeutic agents through alteration of miR-122-target gene expression in these cells [84]. As a target of miR-122, IGF-IR, a receptor tyrosine kinase, plays a critical role in cancer development and progression. Similarly, miR-7 and miR-320 play a tumour suppressor role by downregulating the metabolic IGF-IR/Akt and PI3K/Akt/mTOR signaling pathways. miR-7 overexpression reverses the invasion, migration and metastasis of cancers as a consequence of suppressed glucose metabolism [85,86]. Activated IGF-IR may initiate a signaling cascade axe, the PI3K/AKT signaling pathway, which can induce epithelial-mesenchymal transition (EMT), an important process toward cancer metastasis [87]. Mechanistically, miR-122 disrupts metastasis or EMT process by inhibiting the IGF-IR/PI3K/AKT pathway [88].

Inhibition of IGF-IR phosphorylation was found *in vitro* and *in vivo* by metformin treatment, which is a commonly-used oral anti-hyperglycaemic agent of the biguanide family. Metformin has been reported to inhibit tumour progression of cancer cells by inhibiting mTOR via AMPK [89]. Recent evidence shows that metformin can exert an anticancer effect by suppressing cell cycle through miRNAs modulation [90,91]. In pancreatic cancer cells, metformin induces the expression of miR-26a, miR-192 and let-7c [92], associated with the inhibition of cell proliferation, invasion and increased cell death by targeting HMGA1 (high-mobility group protein HMG-I). In addition, metformin modulates the miR-200 and miR-205 family, which are involved in senescent response to doxorubicin treatment [93]. Notably, miRNA modulation by metformin occurs through the regulation of DICER expression [94]. Therefore, the regulation of the DICER-miRNA axis may represent a novel strategy to treat recurrent or drug-resistance cancer, also in combination with conventional therapies [95].

The miRNAs involved in regulating signaling pathways linked to metabolism in cancer are shown in Table 1.

## 3. miRNAs as Cancer Stroma Messengers Regulating Cancer Metabolism and Tumorigenesis

Much of the cellular heterogeneity within tumours is found in their stromal counterparts. Communication between stromal and tumour cells initiates tumour growth, angiogenesis, invasion and metastasis. Stromal cells include cancer-associated fibroblasts (CAFs), tumour-associated macrophages, pericytes, endothelial cells and infiltrating immune cells. Through specific communications with cancer cells, CAFs directly promote tumour initiation, progression and metastasis. Although the majority of reported tumour-stroma interactions are mediated by secreted soluble factors, emerging evidence indicates that there is yet another type of tumour-stroma communication mediated by secreted vesicles [96], and miRNAs are selectively enriched in these vesicles. Cell-to-cell communications, mediated by the delivery of miRNAs contained in exosomes, is implicated in physiological and pathological processes [97].

CAFs provide critical metabolites for tumour growth and undergo metabolic reprogramming to support glycolytic phenotype, increasing their glucose upload and their delivery of lactate [98]. CAFs undergo a metabolic rearranging, increasing also the production of ketone bodies and glutamine. This metabolic reprogramming of CAFs is mainly driven by the activation of miRNAs. miR-424 downregulates IDH3a during CAF formation. Downregulated IDH3a reduces the level of α-ketoglutarate (α-KG), resulting in the inhibition of proline hydroxylase (PHD) and consequent HIF1 stabilization. Activated HIF1 promotes glycolysis and inhibited OXPHOS [99]. In certain conditions, the pseudo-hypoxic activation of HIF1 is linked to nuclear localization of PKM2, a metabolic enzyme involved in the Warburg behaviour. PKM2 migrates into the nucleus and associates with HIF1, converting its transcriptional activity toward the regulation of OXPHOS. PKM2 and HIF1 associate with embryo-chondrocyte expressed gene-1, a transcriptional repressor allowing downregulation of miR-205. PKM2, HIF1 and miR-205 are the molecular linkers between metabolic and motile reprogramming of cancer cells upon contact with their surrounding CAFs. miRNAs are also involved in the regulation of the Warburg effect of CAFs; miR-210 and miR-186 have been reported to regulate HIF-1 and GLUT-1, respectively (reviewed by Chiarugi and Cirri [5]). Likewise, a pro-inflammatory microenvironment has been linked to the conversion of cancer toward an OXPHOS phenotype. The NF-κB (nuclear factor kappa-light-chain-enhancer of activated B cells), a transcription factor involved in the inflammatory processes, is able to control energy homeostasis and metabolic adaptation to glucose starvation by upregulating mitochondrial function [100].

Inflammation and aberrant immune response are common features of the tumour microenvironment and have been shown to be effectors of tumour-promoting events. Tumour stroma is infiltrated by immune cells, and CAFs actively contribute to maintain chronic inflammation [101]. For instance, inflammation is one of the downstream mechanisms linking hepatocyte nuclear factor 4α (HNF4α) to hepatocellular carcinogenesis. A microRNA-inflammatory feedback loop circuit consisting of miR-124, IL6R, STAT3, miR-24 and miR-629 inhibits HNF4α, resulting in hepatocellular transformation. Once activated, the circuit maintains suppression of HNF4α to sustain cancer development [102]. Another miRNA that links inflammation and cancer metabolism is represented by miR-155. miR-155 is a prominent oncomiR that plays an important role in the metabolically-driven oncogenic process in cancers [103]. Cytokines released during inflammation have been shown to upregulate the glycolysis by the miR-155/miR-143 cascade in breast cancer cells [104]. An inflammation-induced increase in miR-155 levels represses miR-143, which negatively regulates HK2, by targeting the transcriptional activator CCAAT/enhancer binding protein beta (C/EBPβ) and also enhancing HK2 transcription, thereby activating pro-tumourigenic inflammatory STAT signaling [105].

Endothelial cells are the only type of cells present in the tumour microenvironment shown to be actively targeted *in vivo* for miRNA delivery. During cancer progression, an imbalance builds up between angiogenic factors regulating the growth of tumours and their metastasis spread. The altered angiogenic phenotype is mirrored by an aberrant miRNA profile mediated by metabolic re-programming. Extracellular vesicles (EVs) secreted from cells have been found to mediate signal transduction between cells, and EVs are classified, based on their origin, as exosomes, microvesicles and apoptotic bodies. Exosomes produced by cancer cells contain miRNAs related to angiogenesis, the immune system, stroma cell activation and metastasis. So far, miR-122, miR-105, miR-150, miR-10b and miR-126, have been reported to be delivered to the surrounding tumour milieu [105,106,107,108,109]. miR-122 is enclosed into vesicles and delivered to stromal cells in order to reprogram their metabolism. Hence, miR-122, uptaken by surrounding cells, targets PKM2 and represses glycolytic metabolism, thereby lowering glucose utilization by niche cells and allowing glucose exploitation by growing cancer cells [106]. Therefore, by changing glucose consumption by niche cells, cancer-delivered extracellular miR-122 reprograms energy demands to induce cancer progression. In addition, miR-105 secreted by cancer cells into EVs is able to reprogram endothelial cells and tumour-associated macrophages to increase their production of VEGF, thereby facilitating intravasation and metastasis dissemination [108]. In the tumour microenvironment, VEGF released from cancer cells plays a key role in promoting tumour angiogenesis. Recently, increasing attention has been paid to the role of exosomal miRNAs (exo-miRs) related to angiogenesis control that are translated into protein in recipient cells. miR-17-92 and miR-192 inhibited the progression of cancers via suppressing tumour angiogenesis through targeting multiple tumour angiogenesis-inducing genes, TGFBR2 (transforming growth factor, beta receptor 2), HIF1α and VEGFA *in vivo* and *in vitro* [110,111]. Additionally, both miR-29a and miR-29c directly target the 3′UTR of VEGF mRNA, thus inhibiting growth and angiogenesis in tumours implanted in the mice [112]. A recent study showed that miR-206 transfection significantly downregulated VEGF expression in lung cancer cells [113].

During cancer progression, miR-126 is downregulated, with ensuing changes in the expression and activity of metabolism-related factors [77,114,115]. miR-126 is an endothelial-specific species that is essential for maintaining vessel integrity during development. Its role in tumour angiogenesis within cancer stroma is unclear. Huang and colleagues studied the temporal and spatial expression and the role of miR-126 in the course of cervical carcinogenesis, where ‘cross-talk’ of cervical cancer cells and the associated fibroblasts induced downregulation of miR-126 in human umbilical vein endothelial cells (HUVEC), with a consequent increase of tube formation. This study suggests a cancer-stroma cross-talk that induces repression of miR-126 and upregulation of the pro-angiogenic gene adrenomedullin (ADM), and probably also other pro-angiogenic factors, to facilitate angiogenesis and invasive growth of cervical cancer [78]. Exogenous addition of miR-126 precursor suppressed angiogenesis and tumour growth.

In a similar manner, ectopic miR-126 induces the loss of malignancy and the failure of malignant mesothelioma (MM) cells to initiate tumours [77]. miR-126 negatively regulates IRS1 [77,116,117], an adaptor protein mediating IGF-I/insulin signaling, which is involved in various pathological processes [118]. Concurrently, miR-145, miR-128, miR200c and miR-1225-5p target IRS-1 [119,120,121,122].

IRS1 activated by IGF-IR recruits intracellular proteins containing SH2 domains, leading to activation of the PI3K-AKT, which is responsible for most of the metabolic actions of insulin, FOXO1 pathways [123]. FOXO1 regulates the expression of stress-responsive genes and genes involved in cell metabolism and proliferation [124]. Consistent with this, miR-126 induced nuclear translocation of FOXO1 in both non-malignant mesothelial and MM cells, resulting in increased expression of genes involved in the regulation of glucose metabolism and mitochondrial function [77]. Upregulation of miR-126 in MM cells decreased the ACL activity, inducing citrate accumulation in the cytoplasm and stabilisation of HIF-1, which is a critical transcription factor in various cellular and physiologic processes, as it can facilitate the adaption of tumour cells to hypoxia by the activation of the transcription of downstream target genes and by regulation of multiple aspects of tumorigenesis. These include cell proliferation, survival, differentiation, apoptosis and angiogenesis [125]. Accumulation of citrate induces HIF-mediated VEGF-A expression in MM cells, which can be inhibited in the presence of miR-126, being one of its target [77].

ACL links glucose to lipid metabolism and is responsible for the conversion of citrate to cytosolic AcCoA, an important component of several biosynthetic pathways. AcCoA is the substrate for *de novo* synthesis of lipids and protein acetylation. In cancer cells, the majority of AcCoA derives from pyruvate via PDH [126]. Therefore, mitochondrial activity through the pyruvate-citrate shuttle is a critical step for the biosynthesis of FAs and cholesterol, as well as protein acetylation. PDH flux is regulated by cyclic phosphorylation and dephosphorylation of specific PDKs and pyruvate dehydrogenase phosphatases (PDPs), whose function is regulated by cellular nutrients. miR-126 reduced PDK expression, but paradoxically inhibited PDH activity, leading to pyruvate accumulation in the cytosol [127]. Under these conditions, total glucose oxidation by the TCA cycle is rather low, and the energy demand is primarily met by FA and ketone body oxidation.

FAs are enzymatically metabolised into mitochondria through β-oxidation to sustain cellular energy levels during nutrient stress [128]. When cytoplasmic FA levels become too high, FA toxicity ensues, leading to membrane permeability and mitochondrial dysfunction [129]. It is thought that FAs are stored in LDs for energy generation [130], for utilization as membrane building blocks [131] and for biosynthesis of steroid hormones [132].

**Figure 2:**
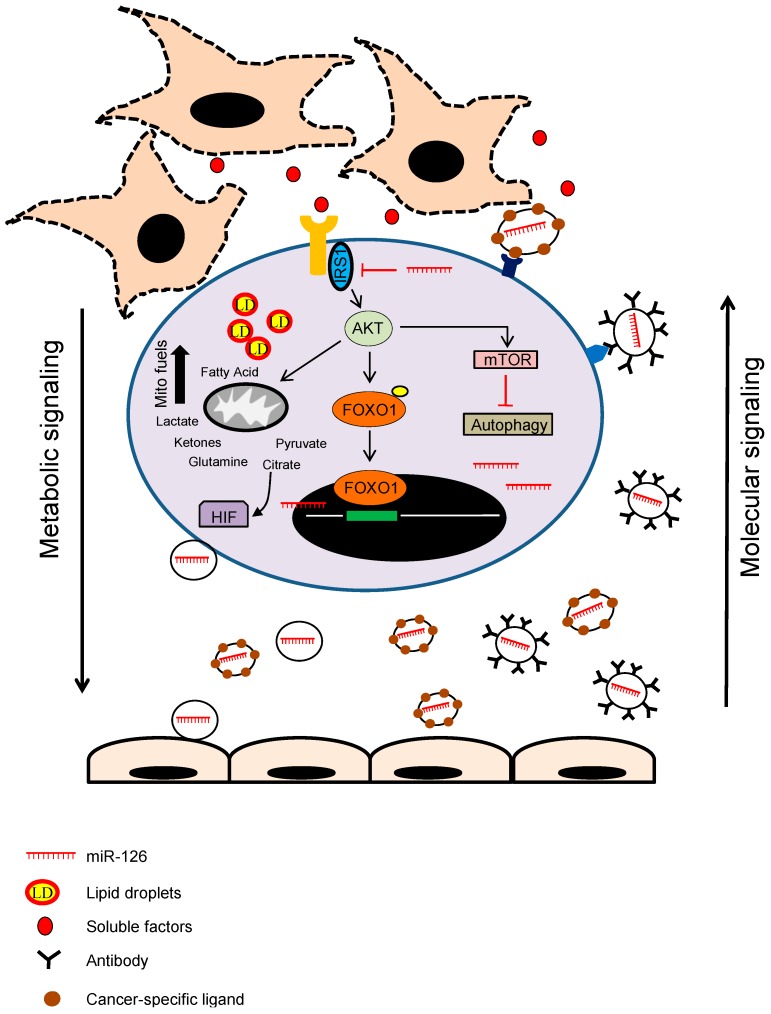
Cross-talk between cancer cells and cancer-associated fibroblasts (CAFs) in the regulation of miRNA expression. In the tumour microenvironment, the cross-talk between the carcinoma cells and CAFs yields soluble factors inducing downregulation of endothelial cell-specific miRNAs, such as miR-126, with resulting induction of proliferation, angiogenesis and malignancy. Restoration of miR-126 inhibits the IRS-AKT pathway, thus initiating a metabolic programme leading to high autophagic flux and HIF1α stabilisation, plus angiogenesis inhibition incompatible with tumour progression.

Many types of cellular stresses, including inflammation and oxidative stress, can induce LD biogenesis [133]. Autophagy promotes lipid build-up in LDs and replenishing LDs with new FA that then move into mitochondria. When needed, LDs efficiently supply FAs for mitochondrial β-oxidation. This cellular adaptation requires activation of the energy sensor AMPK, which in response to energy depletion simultaneously increases LD mobilisation and activates mitochondria [44]. Defects in mitochondria cause massive alterations in cellular FA “routing”: non-metabolised FAs are re-directed to and stored in LDs or expelled from the cell. miR-126 affects mitochondrial metabolism and stimulates LD accumulation in a HIF1α-dependent manner. miR-126 initiates a metabolic programme leading to high autophagic flux and HIF1α stabilisation, incompatible with the progression of MM. miR126-expressing MM cells injected into immunocompromised mice failed to progress beyond the initial stage of tumour formation, showing that increased autophagy has a protective role in MM [77,127] (Figure 2).

## 4. Conclusions

Accumulating evidence indicates that miRNAs play a critical role in regulating many cellular processes, which are often implicated in health and disease, including cancer. The complexity of solid tumours and their distinct pathophysiology relies on interactive communication between various cell types in the neoplastic tissue. Recently, miRNAs have been identified in secreted exosomes. Tumour-derived exosomal miRNAs modulate gene expression of neighbouring or distant recipient cells through paracrine/autocrine signaling. Mechanistically, it is believed that miRNAs themselves exert “sponge-like” effects on various miRNAs, which subsequently inhibits miRNA-mediated metabolic functions. Understanding how such miRNA-mediated communication occurs may eventually lead to opening novel avenues for therapeutic exploitation and/or intervention, particularly for hard-to-treat cancers.

## Figures and Tables

**Table 1:** miRNAs regulating signaling pathways.

miRNA	Target Gene	Function	Cancer Hallmarks	Ref.
**miR-199a**	HIF1α	HIF inhibition	Hypoxia	[47]
**miR-92-1**	VHL	HIF stabilization	Hypoxia	[48]
**miR-20a/b**	VHL	HIF stabilization	Hypoxia	[49]
**miR-21**	VHL	HIF stabilization	Hypoxia	[49]
**miR-22**	VHL	HIF stabilization	Hypoxia	[49]
**miR-101**	VHL	HIF stabilization	Hypoxia	[49]
**miR-106a/b**	VHL	HIF stabilization	Hypoxia	[49]
**miR-150**	VHL	HIF stabilization	Hypoxia	[49]
**miR-200b**	VHL	HIF stabilization	Hypoxia	[49]
**miR-424**	Cullin-2	HIF stabilization	Hypoxia	[50]
**miR-210**	GPD1L	HIF stabilization	Hypoxia	[51]
**miR-23a/b**	GLS	Glutaminolysis inhibition	Metabolism	[52]
**Let-7a**	c-MYC	MYC pathway	Insulin pathway	[53]
	RAS	PI3K/Akt signaling		[54]
	p53	p53 signaling		[22]
	IGF-IR	Insulin/IGF-IR		[80]
**miR-9**	E-cadherin	Induced by c-MYC	Insulin pathway	[55]
**miR-34**	LDHA, MYC, SIRT1	p53 signaling	Apoptosis	[57]
**miR-194/miR-215**		p53 signaling	Apoptosis/cell cycle arrest	[56]
**miR-107**	CDKN1A/p21, HIF	p53 signaling	Hypoxia	[22]
**miR-17-92**	KRAS	Suppressed by p53	Proliferation	[58]
**miR-16-1**	CDK6	Suppressed by p53	Cell cycle arrest	[59]
**miR-143**		Suppressed by p53		[59]
**miR-145**		Suppressed by p53		[59]
**miR-25**	p53	Suppress p53 signaling	Proliferation/cell cycle arrest	[60]
**miR-30d**	p53	Suppress p53 signaling		[60]
**miR-504**	p53	Suppress p53 signaling		[60]
**miR-125b**	p53	Suppress p53 signaling		[60]
**miR-372**	p53	Suppress p53 signaling		[61]
**miR-373**	p53	Suppress p53 signaling		[61]
**miR-326**	PKM2	AMPK signaling	Metabolism	[62]
**miR-451**	CAB9/miR-195	LKB1/AMPK signaling	Proliferation	[63]
**miR-33a/b**	AMPK, IRS2	AMPK/FOXO1 signaling	Glucose metabolism	[38]
**miR-21**	PTEN, HIF, VEGF	PI3K/Akt activation	Proliferation/angiogenesis	[65]
**miR-337**	PTEN	PI3K/Akt signaling		[66]
**miR-543**	PTEN	PI3K/Akt signaling		[67]
**miR-214**	PTEN	PI3K/Akt signaling		[68]
**miR-130**	PTEN	PI3K/Akt signaling		[69]
**miR-181**	PTEN	PI3K/Akt signaling		[70]
**miR-221/222**	PTEN	PI3K/Akt signaling		[75]
**miR-26a**	PTEN	PI3K/Akt signaling	Metastasis/angiogenesis	[71]
**miR-144**	mTOR	PI3K/Akt/mTOR signaling	Proliferation/cell cycle arrest	[73]
**miR-126**	IRS1, VEGF	PI3K/Akt/mTOR signaling	Proliferation/angiogenesis	[76,77,78]
**miR-7**	IGF-IR	Insulin/IGF-IR signaling	Insulin secretion	[79,85]
**miR-375**	IGF-IR	Insulin/IGF-IR signaling		[79]
**let-7**	IGF-IR	Insulin/IGF-IR signaling		[79]
**miR-122**	IGF-IR	IGF-IR/PI3K/Akt	metastasis	[41,80]
**miR-320**	IGF-IR	Insulin/IGF-IR signaling		[86]

## Abbreviations

AcCoA: acetyl-CoA; ACL: ATP citrate lyase; ADM: adrenomedullin; AMPK: AMP-activated protein kinase; CAB9: calcium binding protein 9; C/EBPβ: CCAAT/enhancer binding protein beta; COX10: cytochrome c oxidase 10; DAG: diacylglycerol; DGAT: diacylglycerol acyltransferase; EC: endothelial cells; EMT: epithelial-mesenchymal transition; ER: endoplasmic reticulum; ERRγ: estrogen-related receptor γ; ETC: electron transfer chain; exo-miRs: exosomal miRNAs; EVs: extracellular vesicles; GABPA: GA binding protein transcription factor, α subunit; FA: fatty acid; FAO: fatty acid oxidation; FOXO1: forkhead box protein O1; GLS: glutaminase; GLUT: glucose transporters; GPD2: glycerol-3-phosphate dehydrogenase-2; GPD1L: glycerol-3-phosphate dehydrogenase 1-like; GSH: glutathione; HCC: hepatocellular carcinoma; HIF: hypoxia-inducible factor; HK2: hexokinase 2; HMGA1: high-mobility group protein HMG-I; HUVEC: human umbilical vein endothelial cells; α-KG: α-ketoglutarate; IDH1/2: isocitrate dehydrogenase; ISCU: iron-sulphur cluster assembly proteins; IGF-I: insulin growth factor-I; IGF-IR: insulin-like growth factor-I receptor; IIS: insulin/IGF-IR signaling; IRS: insulin receptor substrate; LDs: lipid droplets; LDHA: lactate dehydrogenase A; LPA: lysophosphatidic acid; miRNAs: microRNAs; mtDNA: mitochondrial DNA; NAFLD: non-alcoholic fatty liver diseases; NF-kB: nuclear factor kappa-light-chain-enhancer of activated B cells; OAA: oxaloacetate; OXPHOS: oxidative phosphorylation; PA: phosphatidic acid; PanK: pantothenate kinase; PDH: pyruvate dehydrogenase; PDK1: pyruvate dehydrogenase kinase 1; PDPs: pyruvate dehydrogenase phosphatases; PHD: proline hydroxylase; PGC-1β: peroxisome proliferator-activated receptor γ, co-activator 1-β; PKM: pyruvate kinase M; SDHD: succinate dehydrogenase, subunit D; SIRT1: sirtuin-1; TAG: triacylglycerol; TCA: tricarboxylic acid; TGFBR2: transforming growth factor, beta receptor 2; VEGF: vascular endothelial growth factor; VHL: von Hippel–Lindau.
